# Isolated central nervous system Aspergillosis infection in a chronic lymphocytic leukemia patient on Ibrutinib: A case report

**DOI:** 10.1186/s12879-020-4894-2

**Published:** 2020-02-24

**Authors:** Thuy-Hong Le, Vikas Kumar, Khubaib Gondal, Martin Barnes, Haseeb Siddique, Barjinder Buttar, Alan Kaell

**Affiliations:** Donald and Barbara Zucker School of Medicine at Hofstra/Northwell, Port Jefferson, USA

**Keywords:** Aspergillosis, Ibrutinib, Chronic lymphocytic leukemia, Aspergillus, Case report

## Abstract

**Background:**

In patients at high risk of opportunistic infections who present with isolated.

neurological symptoms, it is lifesaving to consider Central Nervous System Aspergillosis (CNS-A). Ibrutinib use in chronic lymphocytic leukemia (CLL) has previously been associated with CNS-A. We provide a case report of a patient that presented with primary CNS-A on Ibrutinib therapy without any prior pulmonary or local paranasal signs of infection.

**Case presentation:**

74-year-old Caucasian male with CLL and no prior chemotherapy on ibrutinib for 6 months presented with three months of unsteady gait, occipital headache, and confusion. He has a history of pulmonary sarcoidosis on chronic prednisone 5 mg daily and chronic obstructive pulmonary disease (COPD). He was found to have a “brain abscess” on imaging. Emergent craniotomy confirmed Aspergillus and patient was treated with Voriconazole for 6 months. At six-month follow up, repeat magnetic resonance imaging (MRI) confirmed complete resolution of CNS lesion.

**Conclusions:**

Our case reinforces the importance of being vigilant for isolated CNS-A in CLL patients on ibrutinib who present with neurological symptoms and signs, without prior or co-infection of sino-pulmonary tissue.

## Background

Invasive Aspergillosis (IA) is an opportunistic infection caused by the fungus *Aspergillus* [1]. Immunocompromised patients are at risk; however, in patients with Chronic Lymphocytic Leukemia (CLL) per se are not thought to be at a higher risk of IA. A 2016 correspondence in New England Journal of Medicine (NEJM) alerted clinicians to be aware of the possibility that ibrutinib use in CLL, even without neutropenia, is rarely associated with CNS-A [2]. Either the lung or paranasal sinuses are typically infected prior to or concomitant with CNS-A and therefore may serve as an etiological clinical clue to the correct diagnosis.

The diagnosis of isolated CNS-A is challenging. Neurological symptoms and signs are variable and an intracranial mass, detected by Computed Tomography (CT) or MRI, is not pathognomonic and easily misattributed to malignancy or abscess. Criteria for proven invasive fungal disease is by tissue biopsy histopathology and culture [3], but may not be possible or deemed safe to accomplish. Early diagnosis and prompt surgical and medical treatment reduces morbidity and mortality. Treatment of CNS-A is surgical excision (resection or removal if possible) of the *Aspergillus* granuloma and necrotic tissue treatment with anti-aspergillus therapy [4]. Current Infectious Diseases Society of America (IDSA) guidelines recommend voriconazole as the preferred first line anti-aspergillus therapy [5]. Amphotericin B may also be considered in patients intolerant to voriconzole [5].

## Case presentation

**Fig. 1:**
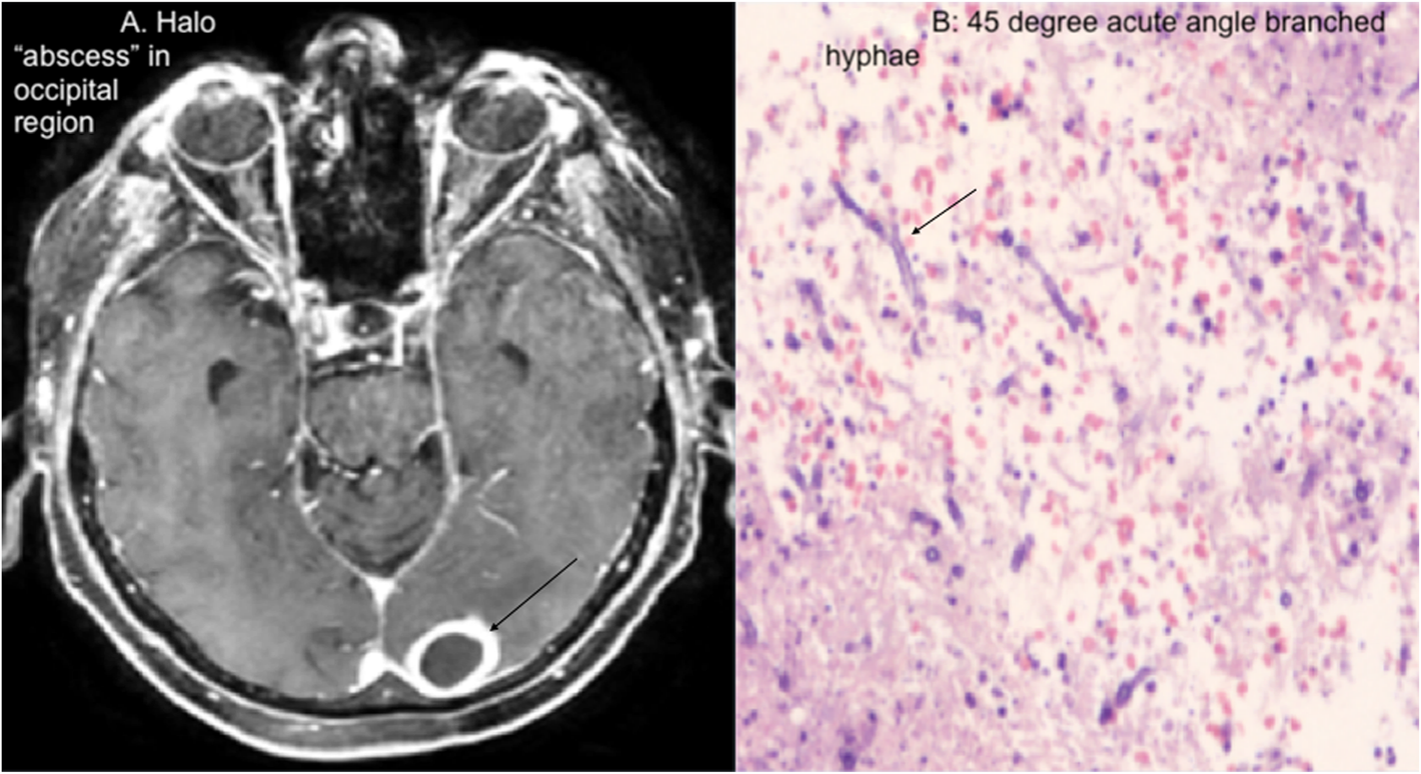
**a** “Halo abscess” in occipital region. **b** “45-degree acute angle branched hyphae

A 74-year-old Caucasian male with CLL and no prior chemotherapy on ibrutinib for 6 months, presented with 3 months of fluctuating, slowly progressive imbalance, unsteady gait, left occipital headache and 1 week of intermittent confusion prior to neurological evaluation. Medical history included: COPD, non-valvular atrial fibrillation (novel oral anticoagulant treated), and pulmonary sarcoidosis on chronic prednisone 5 mg daily. Neurological exam revealed a right upper hemianopsia, unsteady wide based gait. He was afebrile and alert, but mildly confused. Sino- pulmonary exam was normal and no lymphadenopathy or hepatosplenomegaly detected. Basic lab work showed no neutropenia. Head MRI showed a ring enhancing lesion (Fig. 1a) in the left occipital region without sinus abnormalities. An emergent craniotomy to evacuate the brain “abscess” revealed acute 45-degree angle branching hyphae (Fig. 1b), consistent with Aspergillus and confirmed with silver stain and positive culture. Chest CT was without signs of pulmonary aspergillosis or pneumonia and blood cultures were without growth. Transthoracic Echocardiogram performed showed no definitive vegetations. Serum galactomannan levels were not drawn due to lab limitations; however, B-D Glucan levels were positive. Within 72 h of postoperatively initiated voriconazole, his neurological symptoms dramatically improved. At six-month follow-up, repeat MRI confirmed complete resolution of the central nervous system (CNS) lesion and absence of neurological signs and symptoms.

## Discussion and conclusions

Our patient on ibrutinib for CLL and low dose steroids for pulmonary sarcoidosis presented with subacute, neurological symptoms and signs attributed to isolated CNS-A. Aggressive diagnostic surgical and medical treatment led to complete cure.

Case reports predominate the literature. A 2012 meta-analysis compared cases of CNS-A at Massachusetts General Hospital (MGH) (*n* = 14) with globally published cases (*n* = 123) from 2000 to 2011 [6]. Of the 14 cases at MGH, none had isolated CNS-A (only 1 had primary discitis) without antecedent pulmonary or paranasal sinus infection. Of the 123 global cases, 22.8% (*n* = 28/123) did not have an apparent site of extra-CNS infection^.^ Hematogenous dissemination was confirmed from a pulmonary focus in 26.8% (*n* = 33/123) of cases and direct extension from paranasal sinus infection in 27.6% (*n* = 34/123) of cases. None had CLL or ibrutinib exposure [6].

Isolated CNS-A cases are rarely documented in CLL patients [7]. Three CNS-A cases were reported in 2016 from the international RESONATE-2 trial, a prospective observational cohort study of 1149 CLL patients in the United States on ibrutinib therapy. All three patients were on steroids and developed CNS-A within 2 months of initiating Ibrutinib [2]. Our case reinforces the importance of being vigilant for isolated CNS-A in CLL patients on ibrutinib who present with neurological symptoms and signs, even without prior or co-infection of sino-pulmonary tissue, as early recognition and treatment can be lifesaving. Prompt neuroimaging prudent to identify if a CNS space occupying lesion was accessible to biopsy.

Management included surgical evacuation with an attempt to remove or at least reduce the infection and voriconazole for 6 months, consistent with IDSA guidelines [5]. Due to limited access to serum voriconazole level testing and slow turnaround time, serum levels were unable to be monitored. However, adequate CNS penetration was clinically documented by absence of neurological signs and symptoms on physical exam with resolution of CNS lesion on MRI at follow-up.

## Abbreviations

CLL: Chronic lymphocytic leukemia
CNS-A: Central nervous system aspergillosis
COPD: chronic obstructive pulmonary disease
CT: Computer tomography
IA: Invasive aspergillosis
IDA: Infectious disease society of America
MRI: Magnetic resonance imaging
NEJM: New England journal of medicine

## References

[1] About Aspergillosis. Centers for Disease Control and Prevention. 2019. https://www.cdc.gov/fungal/diseases/aspergillosis/definition.html.

[2] Sharman J (2016). Ibrutinib for chronic lymphocytic leukemia: NEJM. N Engl J Med.

[3] Peter Donnelly J, Chen SC, Kauffman CA (2019). Revision and update of the consensus definitions of invasive fungal disease from the European Organization for Research and Treatment of Cancer and the mycoses study group education and research consortium. Clin Infect Dis.

[4] Nadkarni T, Goel A (2005). Aspergilloma of the brain: an overview. J Postgrad Med.

[5] Patterson TF, Thompson GR (2016). Practice guidelines for the diagnosis and Management of Aspergillosis: 2016 update by the infectious diseases society of America. Clin Infect Dis.

[6] Kourkoumpetis TK (2012). Central nervous system Aspergillosis: A series of 14 cases from a general hospital and review of 123 cases from the literature. Medicine.

[7] Payot A (1999). Primary central nervous system Aspergillosis: a case report and review of the literature. Clinical Microbiology and Infection, ESCMID.

